# Correlation Analysis of PM_10_ and the Incidence of Lung Cancer in Nanchang, China

**DOI:** 10.3390/ijerph14101253

**Published:** 2017-10-19

**Authors:** Yi Zhou, Lianshui Li, Lei Hu

**Affiliations:** 1 College of Applied Meteorology, Nanjing University of Information Science and Technology, Nanjing 210044, China; 2 College of Economics and Management, Nanjing University of Information Science and Technology, Nanjing 210044, China; llsh@nuist.edu.cn; 3 Agrometeorological Experiment Station of Jiangxi Province, Nanchang 330200, China; alei8030@sina.com

**Keywords:** PM_10_, air pollution, lung cancer, grey correlation, data envelopment analysis

## Abstract

Air pollution and lung cancer are closely related. In 2013, the World Health Organization listed outdoor air pollution as carcinogenic and regarded it as the most widespread carcinogen that humans are currently exposed to. Here, grey correlation and data envelopment analysis methods are used to determine the pollution factors causing lung cancer among residents in Nanchang, China, and identify population segments which are more susceptible to air pollution. This study shows that particulate matter with particle sizes below 10 micron (PM_10_) is most closely related to the incidence of lung cancer among air pollution factors including annual mean concentrations of SO_2_, NO_2_, PM_10_, annual haze days, and annual mean Air Pollution Index/Air Quality Index (API/AQI). Air pollution has a greater impact on urban inhabitants as compared to rural inhabitants. When gender differences are considered, women are more likely to develop lung cancer due to air pollution. Smokers are more likely to suffer from lung cancer. These results provide a reference for the government to formulate policies to reduce air pollutant emissions and strengthen anti-smoking measures.

## 1. Introduction

Air pollution has a serious impact on human health, in particular the incidence of lung cancer, which is the most common cancer in the world. In 2013, the World Health Organization (WHO) classified outdoor air pollution as carcinogenic to humans (International Agency for Research on Cancer, Group 1) [1]. Residents exposed to air pollution have shown higher frequencies of mutations in cells, DNA damage and other chromosomal aberrations which lead to increasing the incidence of lung cancer and polycyclic aromatic hydrocarbons (PAHs) is the most important pathogenic factor causing these lesions [2,3]. Exposure to PM_10_, SO_2_, and NO_2_ significantly increase the risk of lung cancer [4]. As a developing country, China has the world’s most serious burden of lung cancer[5], incidence rate of lung cancer was 50.9 per 100,000 for men and 22.4 per 100,000 for women in 2015 [6]. Therefore, knowing which pollutant among air pollution factors is most closely related to lung cancer is of great significance.

Scholars have studied the relationship between components of air pollution and lung cancer in many regions of the world. For example, Raaschou-Nielsen et al. [7] found that between 1985 and 2005, sulfur and nickel pollutants had the most significant effect on lung cancer in Europe. Furthermore, Li et al. [8] found that in Hong Kong, automobile exhaust and trace metals accounted for most of the PM_10_ pollution, and caused lung cancer. Finally, Guo et al. [9] determined that in China, PM_2.5_ and O_3_ both had a significant statistic relation with the incidence of lung cancer. 

Many studies have explored the potential health risks of PM_10_ among residents in China. An et al. [10] found that in 2009, most of China’s residents were exposed to air with an average annual PM_10_ concentration of 40–100 μg/m^3^. However, the PM_10_ concentration safe limit suggested by WHO is 20 μg/m^3^. Shen et al. [11] found that the majority of the population in Henan Province, China were exposed to toxic air while Zhang et al. [12] determined that in Beijing, with more city buildings and denser roads, the health risk to local residents increased as the concentration of PM_10_ increased.

In recent years, rapid economic development has resulted in deleterious health impacts for the residents of many Asian countries. Many of these health impacts have been attributed to elevated concentrations of PM_10_, and hence, there have been several studies focusing on PM_10_ induced mortality rates. Chen et al. [13] found that as the PM_10_ concentration in northern China increased by 10 mg/m^3^, lung cancer mortality increased by 3.4% to 13.6%. Furthermore, Chen et al. [14] found that between 2010 and 2014, increased concentrations of PM_10_ in Chengdu have resulted in increased mortality. Maleki et al. [15] found that between2009 and 2014, high concentrations of PM_10_ caused 3777 deaths in Ahvaz, Iran. Ho et al. [16] determined that during 2012, high concentrations of PM_10_ in Ho Chi Minh City, Vietnam killed 204 residents. Other scholars have focused on the negative effects of increased PM_10_ concentrations on the economy. Hou et al. [17] determined that in 2009, China’s health related economic losses due to PM_10_ reached $U.S. 106.5 billion, approximately equal to 2.1% of China’s Gross Domestic Product (GDP) in that year. Hou et al. [18] also found that from 2008 to 2012, the average annual economic loss associated with PM_10_ pollution was $U.S. 9.5 billion.

Developed nations such as the Unites States and those in Europe have already experienced the industrialization process and suffered from the subsequent severe air pollution brought about by this. Consequently, these regions fully recognize the importance of improving air quality and have adopted various measures to reduce pollutant emissions in order to improve the health of the population. As a result of this process, new industries have developed which have helped to boost economic growth. Keuken et al. [19] found that in Rotterdam, The Netherlands, by applying stringent emission standards to prevent high emitters near residential areas, the concentration of PM_10_ was reduced by 18 μg/m^3^ during the past 24 years, and the average life expectancy of the residents increased by 13 months over the same period. Carugno et al. [20] found that between 2003 and 2014, after implementing air quality control policies such as renewal of vehicle exhaust systems and engines, incentives to purchase hybrid and electric vehicles, the decrease in PM_10_ concentrations in Lombardy, Italy resulted in a reduction in the number of deaths caused by PM_10_; approximately 343 people died during the first 5 years of the study, the number was reduced to 253 between 2007 and 2010, and between 2011 and 2014, the death toll was reduced to 208. Research conducted by Castro et al. [21] showed that by applying national regulations such as fuel requirements and maximum permitted pollution levels, the decrease of PM_10_ concentrations had a positive effect on economic growth, in the Agglomeration of Lausanne-Morges, Switzerland, during 2005 to 2015; health related impacts of the reduction of PM_10_ concentration were monetarized at approximately CHF 36 million annually.

Many scholars have conducted air pollution and lung cancer studies in China, but there are few studies related to the air pollution and population health in the middle reaches of the Yangtze River. The aim of this paper was to understand the relationship between the incidence of lung cancer and air pollution in Nanchang, China and explore the characteristics of lung cancer incidence in different population demographics such as residence, gender and smoking history. Nanchang, the capital city of Jiangxi Province, is one of the central cities of the middle reaches of the Yangtze River and the core city of Poyang Lake eco economic zone. Automobile exhaust, soil dust, coal combustion, building dust and metallurgical dust are the major sources of air pollution in Nanchang. The incidence rate of newly diagnosed lung cancer cases was 55.9 per 100,000 for men and 24.8 per 100,000 for women in 2013 [22]. It is of great practical significance to study the association between air pollution and the incidence of lung cancer, to give scientific countermeasures and suggestions for the protection of public health.

## 2. Materials and Methods

### 2.1. Methods

#### 2.1.1. Air Pollution Index and Air Quality Index

Based on the Environmental Protection Laws of the People’s Republic of China (Standing Committee of the National People's Congress, 2014) [23] and the Law of the People’s Republic of China on the Prevention and Control of Atmospheric Pollution (Standing Committee of the National People's Congress, 2015) [24], China has formulated the Ambient Air Quality Standard. Based on this standard, the air pollution index (API) is suitable for studying short-term air quality trend. API simplifies several air pollutant concentrations in routine monitoring as a single exponential and reveals not only air quality status but also the air pollution situation.

Prior to 2012, China’s air pollution index included SO_2_, NO_2_ and PM_10_. When the concentration of a pollutant is $C_{i,j}\leq C_{i}\leq C_{i,j+1}$, its pollution index is:

(1) $$I_{i}=\frac{\left(C_{i}-C_{i,j}\right)\left(I_{i,j+1}-I_{i,j}\right)}{\left(C_{i,j+1}-C_{i,j}\right)}+I_{i,j}$$

where $I_{i}$ is the pollution index of the *i*-th pollutant, $C_{i}$ is the concentration of the *i*-th pollutant, $I_{i,j}$ is the pollution index value of the *i*-th pollutant at turning point *j*, and $C_{i,j}$ is the concentration of the *i*-th pollutant (for $I_{i,j}$) at turning point *j.* $C_{i,j+1}$ is the concentration of the *i*-th pollutant (for $I_{i,j+1}$) at turning point j+1. The air pollution index, API, is the largest of all the pollution indices:

(2) $$API=\max\left(I_{1},I_{2},\cdots ,I_{i},\cdots ,I_{n}\right)$$

In 2012, executive meetings of the State Council agreed to issue a new revised Ambient Air Quality Standard (GB 3095-2012; Chinese Research Academy of Environmental Sciences, 2012) [25]. At the same time, the Ministry of Environmental Protection of China issued the Technical Specification for Ambient Air Quality Index (for trial implementation) [26] to quantitatively describe air quality. AQI adopts more stringent standards and includes more pollutant indicators such as PM_2.5_, O_3_ and CO; consequently, results evaluated using the AQI better reflect the atmospheric conditions.

With respect to calculating AQI, the concentration limit of each pollutant (individual air quality index, IAQI) were calculated based on actual measured values of pollutant concentrations including fine particulate matter (PM_2.5_), inhalable particulate matter (PM_10_), sulfur dioxide (SO_2_), nitrogen dioxide (NO_2_), ozone (O_3_), and carbon monoxide. IAQI is calculated as follows:

(3) $$IAQI_{P}=\frac{IAQI_{Hi}-IAQI_{Lo}}{BP_{Hi}-BP_{Lo}}\left(C_{p}-BP_{Lo}\right)+IAQI_{Lo}$$

where $IAQI_{P}$ is the air quality index of pollutant P, $C_{p}$ is the mass concentration value of pollutant P, $BP_{Hi}$ is the concentration breakpoint that is no more than $C_{p}$, $BP_{Lo}$ is the concentration breakpoint that is no less than $C_{p}$, $AQI_{Hi}$ is the index breakpoint corresponding to $BP_{Hi}$, and $IAQI_{Lo}$ is the index breakpoint corresponding to $BP_{Lo}$. From the IAQI of each pollutant, the maximum is then determined to be AQI. If the AQI is greater than 50, and the maximum pollutant of IAQI is defined as the primary pollutant then:

(4) $$AQI=\max\left(IAQI_{1},IAQI_{2},IAQI_{3},\cdots ,IAQI_{n}\right)$$

The Environmental Protection Bureau of Nanchang City did not monitoring AQI related data (PM_2.5_, O_3_ and CO) until 2013 under the requirement of Ministry of Environmental Protection of China. Therefore in this paper, the API index was used prior to 2013 due to lack of related pollutant indicators, and the AQI index was used after 2013. Because AQI and API are two different indices, significance test are required to determine whether these two indices can be used simultaneously or not [27,28]. Daily AQI and API were used to conduct significance test from 2013 to 2014. These two indices were statistically significant because the *p*-value of 0.000 was 4.11 × 10^−8^ less than 0.05, indicating that AQI and API were significantly related.

#### 2.1.2. Grey Correlation Analysis

Grey correlation analysis is a quantitative description and comparative method for investigating trends within a system [29,30]. Grey correlation analysis has many advantages: the amount of data required is not large, therefore grey correlation analysis has advantage in dealing with small data samples; a strict calibration method is not required for Grey correlation analysis, so it is simpler and easier to use than other statistical methods; the grey relation results are also more intuitionistic and understandable.

The method works by determining the similarity of the sequence reference data array with comparative data. Grey correlation analysis has been widely used in air quality assessment and prediction. For example, You et al. [31] assessed the air quality in Japan and found that air quality declined year on year from 2008. Furthermore, Qin et al. [32] assessed the winter particulate matter concentration for Beijing, Shanghai, Guangzhou and Lanzhou during 2013 and 2014. Their results showed that particulate matter was highly correlated with CO, NO_2_, and SO_2_. Wang et al. [33] simulated the air pollution of five cities in the Jiangsu Province, China using the improved grey dynamic trend model, which was shown to simulate air pollution with more accuracy. Chen et al. [34] simulated the hourly concentrations of PM_10_ and PM_2.5_ of Taichung in 2008, then compared the results with data obtained from a back-propagation artificial neural network (BPNN). Their results showed that the grey model can predict the hourly PM concentration accurately. Finally, Pan et al. [35] found that the grey correlation model can be used to simulate the air quality in Tianjin during 2001 to 2009, and they predicted that air quality will continue to improve during 2010 to 2015.

Grey correlation analysis was applied following a five-step process:

Step 1: Define the reference and comparison sequences.

The data sequence that reflects the characteristics of a system is the reference sequence and the data sequences that affect the behavior of a system are the comparison sequences.

Let sequence $X_{0}={[x}_{0}\left(1\right),x_{0}\left(2\right),\ldots ,x_{0}\left(n\right)]$ be the reference sequence (1~n: serial number for each sample), and let sequence $X_{i}={[x}_{i}\left(1\right),x_{i}\left(2\right),\ldots ,x_{i}\left(n\right)]$ be the comparison sequences (i=1,2,⋯,m; 1~m: serial number of each influencing factor. 1~n: serial number of each factor).

Step 2: Apply the non-dimensional method to the reference and comparison sequences.

Because the physical meaning and dimensions of each factor in the system differ, it is difficult to draw an accurate conclusion when factors are compared with each other. Thus, when dealing with the grey relational grade analysis, non-dimensionless data processing procedure is usually required as follows:

(5) $$X_{0}\left(j\right)=\frac{x_{0}\left(j\right)-x_{0,\min}}{x_{0,\max}-x_{0,\min}}$$

where *j* is the serial number of samples from the reference sequence, $x_{0,\max}$ is the maximum value of the reference sequence and $x_{0,\min}$ is the minimum value of the reference sequence.

(6) $$X_{ij}=\frac{x_{ij}-x_{ij,\min}}{x_{ij,\max}-x_{ij,\min}}\left(1\leq j\leq m,1\leq j\leq n\right)$$

In Equation (6), $x_{ij}$ is data for the *i*-th factor *j*-th sample in the comparison sequence, $x_{ij,\max}$ is the maximum value of the *i*-th factor sample and $x_{ij,\min}$ is the minimum value of the *i*-th factor sample.

Step 3: Determine the grey correlation coefficient for the reference and comparison sequences.

The degree of correlation refers to the difference between the geometric shapes of the curves for the reference and comparison sequences. As such, the difference between the curves can be used as a measure of the degree of correlation. For reference sequence $X_{0}$ and several comparison sequences, $X_{1},X_{2},\cdots ,X_{n}$, the correlation coefficients of the reference sequence and the comparison sequences at different times are denoted by $\xi \left(X_{i}\right)$. This is called the resolution coefficient, and its values range from 0 to 1; however, its value is usually 0.5:

(7) $$\xi_{i,1}\left(j\right)=\frac{\min_{i}\min_{j}\left|x_{0}\left(j\right)-x_{ij}\right|}{\left|x_{0}\left(j\right)-x_{ij}\right|+\rho \max_{i}\max_{j}\left|x_{0}\left(j\right)-x_{ij}\right|}$$

(8) $$\xi_{i,2}\left(j\right)=\frac{\max_{i}\max_{j}\left|x_{0}\left(j\right)-x_{ij}\right|}{\left|x_{0}\left(j\right)-x_{ij}\right|+\rho \max_{i}\max_{j}\left|x_{0}\left(j\right)-x_{ij}\right|}$$

(9) $$\xi_{i}\left(j\right)=\xi_{i,1}\left(j\right)+\xi_{i,2}\left(j\right),$$

Step 4: Determine the degree of correlation ($r_{i}$).

The correlation coefficient is the degree of relevance between the comparison sequences and the reference sequence at different moments; consequently, it has more than one value. Here, it is necessary to concentrate the correlation coefficients of each point in the curve as an average value, measuring the degree of correlation between the comparison sequence and the reference sequence:

(10) $$r_{i}=\frac{1}{n}\sum_{j=1}^{n}\xi_{i}\left(j\right)\left(i=1,2,\cdots ,m\right)$$

Step 5: Sorting the degree of correlation.

Relevance between factors is described by the order of the degree of correlation, as opposed to the magnitude of the correlation. Therefore, the relational sequence is formed by arranging the degree of correlation for sub sequences to the same generating sequence in order of size, denoted by {x}; this reflects the pros and cons of each subsequence to the generating sequence. While $r_{0}i>r_{0}j$, for the same generating sequence $\left\{x_{0}\right\}$, $\left\{x_{i}\right\}$ is superior to $\left\{x_{j}\right\}$ and is denoted by $\left\{x_{i}\right\}>\left\{x_{j}\right\}$, $r_{0}i$ is the eigenvalue of the i-th subsequence to the generating sequence.

#### 2.1.3. Data Envelopment Analysis

Data envelopment analysis (DEA) is a linear programming methodology to evaluate the efficiency of multiple decision-making units (DMUs) when the production process presents a structure of multiple inputs and outputs [36]. This method is not required to determine the explicit expression of the relationship between input and output variables, which eliminates many subjective factors, and hence, has strong objectivity. Therefore, the DEA method is widely used in efficiency evaluation. Many scholars have used DEA to evaluate environmental pollution, and recommend decision-making plans. Sueyoshi and Yuan [37] determined that the Chinese government should allocate economic resources to cities, strengthen environmental protection, and bring energy consumption under control. In a follow up study, Sueyoshi and Yuan [38] found that industries need to reduce fossil fuel use and use more renewable green energy. Wang et al. [39] determined that China’s industrial zones should reduce pollution through technical investment and reduce the proportion of coal use. Finally, Moutinho et al. [40] argued that, although high environmental taxes and high pressure environmental policies can make European countries livable, these policies can also hamper economic growth.

Data envelopment analysis regards an economic system or a production process as an entity (unit). Within a certain range, units invest a quantity of production factor and produce a quantity of “product”; such units are called DMUs. The same type of DMU, with the same target and task, the same external environment, and the same input and output indicators can form a DMU set. 

The input vector of a DMU in an economic (production) activity is $X=(x_{1},\cdots ,x_{i},\cdots ,x_{m})$, where $x_{i}$ indicates the *i*-th input, the output vector is $Y=(y_{1},\cdots ,y_{r},\cdots ,y_{s})$, $y_{r}$ indicates the *r*-th output;$\left(X_{j},Y_{j}\right)$ is the input and output vector of the *j*-th DMU, and $\left(X_{0},Y_{0}\right)$ are the corresponding indices of the evaluation DMU. Thus, the entire production activity of the DMU can be represented by $\left(X,Y_{}\right)$, and the input set of *n* DMUs can form an n × m order input matrix, while the output set can form an n × m order output matrix.

In this paper, we use the BCC model. The BCC model was proposed by Banker, Charnes and Cooper [41], and is defined as follows:

(11) $$\left\{ \begin{array}{l} \min\left[\theta -\xi \left(e^{T}S^{-}+e^{T}S^{+}\right)\right]\\ s.t.\sum_{j=1}^{n}X_{j}\lambda_{j}+s^{-}=\theta X_{0}\\ \sum_{j=1}^{n}Y_{j}\lambda_{j}-s^{+}=Y_{0}\\ \sum_{j=1}^{n}\lambda_{j}=1_{}\\ \lambda_{j}\geq 0,j=1,2,\cdots ,n.\\ \lambda_{j}\geq 0,s^{+}\geq 0,s^{-}\geq 0,\theta \in E \end{array}\right.$$

The generation possibility set of the BCC model for computing pure technical efficiency can be expressed as:

(12) $$T_{BCC}=\{(X,Y)|X\geq \sum_{j=1}^{n}X_{j}\lambda_{j},Y\leq \sum_{j=1}^{n}Y_{j}\lambda_{j},\lambda \geq 0,j=1,2,\cdots ,n.\}$$

When θ=1, the DMU *j* is technique effective. When θ<1, the DMU *j* is not technique effective.

### 2.2. Data Sources

#### 2.2.1. Meteorological Data

Meteorological observational data from 2003 to 2014 (12 years in total) was provided by the Nanchang National Meteorological Station. According to the Technical Regulation for Haze Pollution Day Judging (on trial, Ministry of Environmental Protection of the People’s Republic of China, 2014) [42], the haze days per year of Nanchang were obtained.

#### 2.2.2. Air Quality (Air Pollutants) Monitoring Data

Air quality data was provided by Environmental Protection Bureau of Nanchang City collected from nine environmental monitoring sites. These monitoring data from 2003 to 2014 (12 years in total) include many air pollution factors such as daily mean PM_10_, NO_2_ and SO_2_. Nanchang began monitoring air quality earlier than most other cities in China. Starting in 2001, Environmental Protection Bureau of Nanchang City and Nanchang Municipal Meteorological Bureau began to work together to set up environmental monitoring sites. The collected data were processed by professional personnel. Therefore, the accuracy, continuity and authority of the data are ensured. 

#### 2.2.3. Lung Cancer Case Data

Lung cancer case data were obtained from a local hospital (the hospital specialises in oncology, it is the research center for cancer prevention and control of Jiangxi Province, a tertiary referral hospital with medical treatment, prevention, teaching and scientific research). Lung cancer case data is in the form of lung cancer registration data includes case number, gender, age, time of diagnosis, place of residence (urban or rural), and smoking history.

#### 2.2.4. Population Statistical Data

Population data for Nanchang during 2003 to 2014 was provided by the Nanchang Statistical Yearbook (Bureau of Statistics of Nanchang, 1996–2014) [43].

## 3. Results

Newly diagnosed lung cancer patients in Nanchang from 2003 to 2014, are shown in Table 1. It is clear that the number of lung cancer patients is on the rise: In 2003, there was 119 cancer patients. In 2012, that number had increased to 454. 

For people living in different parts of Nanchang, the number of rural lung cancer patients are growing much faster than urban lung cancer patients: In 2003, the number of lung cancer in urban areas (99) was approximately five times the incidence of lung cancer in rural areas (20). While in 2014, the number of lung cancer in urban areas (229) and the incidence of lung cancer (225) is almost identical.

The number of male lung cancer patients is far higher than that of female patients; the number of smoking lung cancer patients is far more than that of non-smoker lung cancer patients. 

### 3.1. Grey Correlation Analysis

This section employs the grey correlation analysis method to explore the most serious air pollution factors contributing to lung cancer. The incidence of lung cancer in Nanchang was used as reference sequence, while the air pollution index was used for comparison sequences. There can be a time lag between air pollution and lung cancer, so different time lags were taken into consideration. Specific data used in the grey correlation analysis are shown in Table 2.

#### 3.1.1. Grey Correlation for Accumulated Air Pollution Factors and the Accumulated Incidence of Lung Cancer for Different Time Lags

**Figure 1:**
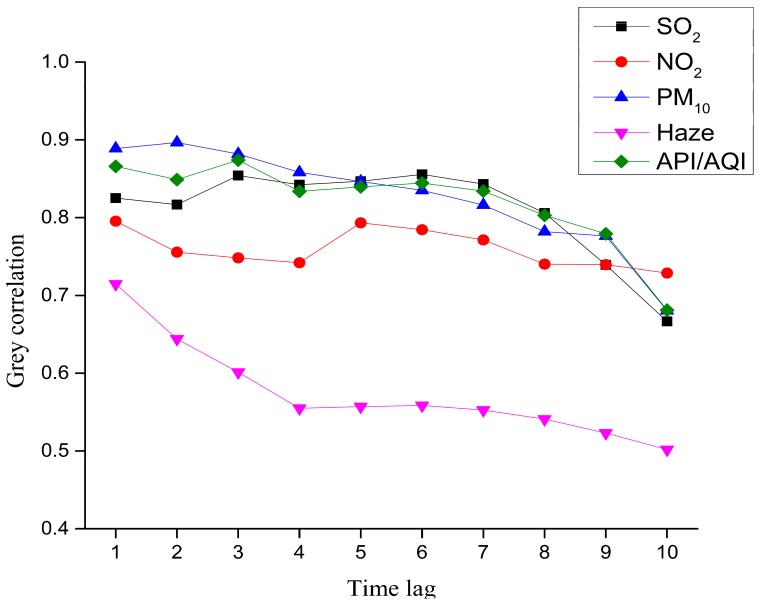
Line graph showing grey correlation for accumulated air pollution factors and accumulated incidence lung cancer for different time lags.

The accumulated incidence of lung cancer in Nanchang was used as a reference sequence (2004–2014, 2005–2014, 2006–2014, 2007–2014, 2008–2014, 2009–2014, 2010–2014, 2011–2014, 2012–2014, 2013–2014). Corresponding to the reference sequence, the accumulated annual mean concentration of SO_2_, NO_2_, PM_10_, annual haze days, and annual mean API/AQI were used as comparison sequences with time lags of between one and ten years (2003–2013, 2003–2012, 2003–2011, 2003–2010, 2003–2009, 2003–2008, 2003–2007, 2003–2006, 2003–2005, 2003–2004). The grey correlation for the respective time lags was then calculated and the results are shown in Table 3 and Figure 1.

When the time lag is between one and four years, the grey correlation for PM_10_ is higher than any other pollution factors, indicating that PM_10_ has the greatest impact on the incidence of lung cancer. For time lags of five, nine and ten years, PM_10_ has the second highest grey correlation, suggesting that PM_10_ also has an impact on the incidence of lung cancer for these time lags. For time lags between one and three years, the grey correlation of API/AQI is the second highest, behind PM_10_. When the time lag is between six and eight years, the API/AQI grey correlation is second only to SO_2_. At a lag of nine years, the grey correlation of API/AQI is the highest, suggesting that the incidence of lung cancer in Nanchang residents was also closely related to API/AQI.

The grey correlation of haze is the lowest in all time lags, meaning that haze does not have the strongest impact on the incidence of lung cancer while comparing with other pollution factors.

In summary, as the time lag increases all the air pollution factors show a decreasing trend with the incidence of lung cancer and PM_10_ appears to be one of the most significant factors influencing the incidence of lung cancer in Nanchang.

Lung cancer is a chronic disease that relies on cumulative effects, therefore the incidence should increase with longer lags, which seems contradictory to our conclusion. However, practical considerations need to be taken into consideration. In China, many government officials are blindly pursuing the rapid growth of GDP, ignore the pollution caused by the introduction of the heavy polluting industries. For Nanchang, the situation is the same. The GDP in Nanchang was 64.1 billion Yuan in 2003. After 2003, GDP annual growth exceeded 10% and annual growth rate of GDP in Nanchang was far higher than the annual growth rate of GDP in China. In 2014, the GDP in Nanchang was 336.7 billion Yuan, over 5 times as much as GDP in 2003 [42]. The rapid growth of GDP brought by heavy pollution industry and surge in car numbers has made air pollution worse year by year. Automobile exhaust and coal combustion contain large number of PAHs. Increased PAHs concentration can significantly increase the incidence of lung cancer [3]. The cumulative amount of air pollution factors that cause lung cancer varies annually, almost increasing year by year, which lead to the increasing number of lung patients that can be seen in Table 2.

#### 3.1.2. Grey Correlation for Air Pollution Factors during a 5-Year Period and the Incidence of Lung Cancer during 5-Year Period with Different Time Lags

The grey correlation for air pollution factors during 5-year periods and the incidence of lung cancer in 5-year periods with different time lags were calculated. Firstly, the incidence of lung cancer in Nanchang during 2010–2014 was used as a reference sequence. Corresponding to the reference sequence, annual mean concentrations of SO_2_, NO_2_, PM_10_, and annual haze days along with annual mean API/AQI were used as comparison sequences with time lags of between one and seven years (2009–2013, 2008–2012, 2007–2011, 2006–2010, 2005–2009, 2004–2008, 2003–2007). The grey correlations for the respective time lags were then calculated and the results are shown in Table 4.

Results show that the grey correlation for PM_10_ is the highest (0.7716) when the time lag is one year and seven years. This indicates that the annual mean concentration of PM_10_ and API/AQI from 2009 to 2013and 2003 to 2007has the most significant impact on the incidence of lung cancer from 2010 to 2014. For lag three, five and six years, the grey correlation for PM_10_ is the second highest. This indicates that the annual mean concentration of PM_10_ from 2007 to 2011, 2005 to 2009 and 2004 to 2008 also has huge impact on the incidence of lung cancer from 2010 to 2014.

Next, the incidence of lung cancer in Nanchang during 2009–2013 was used as a reference sequence. and annual mean concentrations of SO_2_, NO_2_, PM_10_ and annual haze days along with annual mean API/AQI were used as comparison sequences with time lags of between one and six years (2008–2012, 2007–2011, 2006–2010, 2005–2009, 2004–2008, 2003–2007). The grey correlation for the different time lags was calculated and the results are shown in Table 5.

Results show that, with a lag of one, two, three, and six years, PM_10_ has either the highest or second highest grey correlation, indicating that PM_10_ has a significant impact on the incidence of lung cancer during 2009 to 2013. Between a lag of one to six years, the grey correlation for API/AQI is the either the highest or the second highest, suggesting API/AQI also has a major impact on the incidence of lung cancer during 2009 to 2013.

Air pollution factors other than PM_10_ and API/AQI, rarely display high grey correlations. The grey correlation for SO_2_ is the second highest when there is a four year lag and the grey correlation for NO_2_ is the highest when there is a five year lag. The grey correlation for annual haze days never achieves a rank of first or second. Thus, we can infer that SO_2_, NO_2_ and annual haze days do not substantially impact the incidence of lung cancer during 2009 to 2013.

Finally, the incidence of lung cancer in Nanchang during 2008–2012 was used as a reference sequence and annual mean concentrations of SO_2_, NO_2_, PM_10_, haze days, and API/AQI were used as comparison sequences with time lags from between one and five years (2007–2011, 2006–2010, 2005–2009, 2004–2008, 2003–2007). The grey correlation for the different time lags was calculated and the results are shown in Table 6.

At time lags of two, three, and five years, the grey correlation for PM_10_ is the either the highest or the second highest. Furthermore, at time lags of between one and three years, the grey correlation for API/AQI is the either the highest or the second highest. These results suggest that both PM_10_ and API/AQI have a significant impact on the incidence of lung cancer from 2008 to 2012.In summary, our results show that PM_10_ is the most serious air pollution factor causing lung cancer.

### 3.2. DEA

In this section, we use DEA to study the main air pollution factors contributing to lung cancer and study the relationship between the incidence of lung cancer and air pollution factors for different types of patients.

#### 3.2.1. Lung Cancer Incidence and Air Pollution Factors in Nanchang

**Figure 2:**
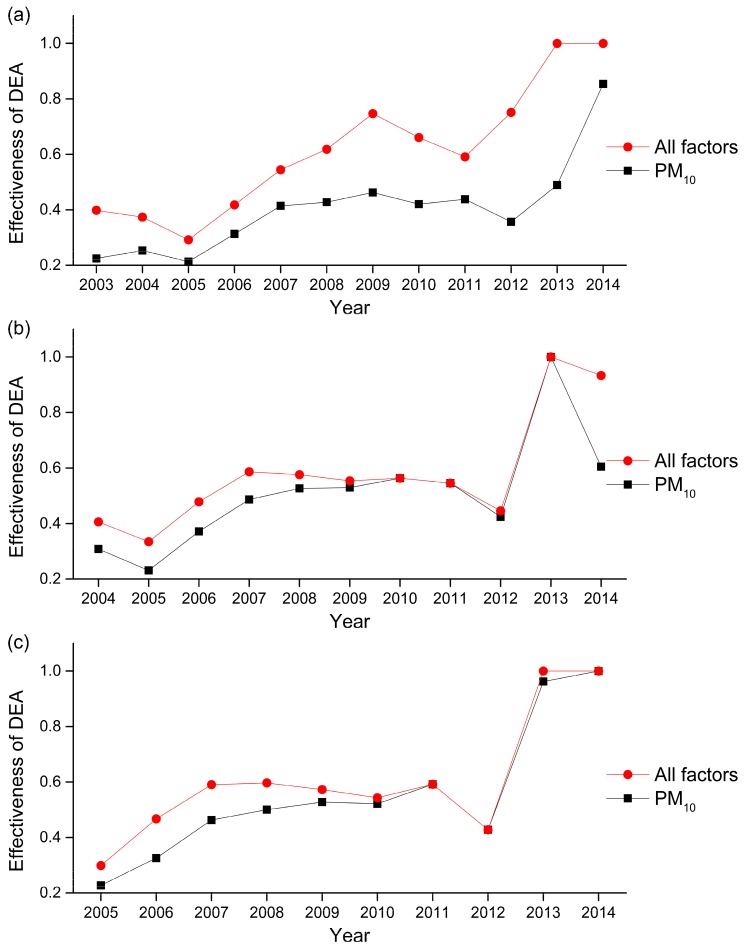

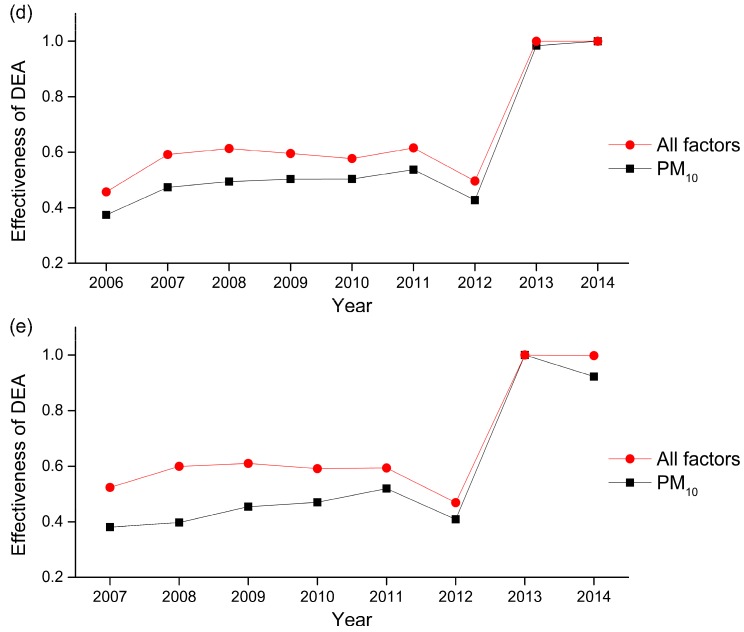
Line graph for Effectiveness of DEA in different time lags between PM_10_ and incidence of lung cancer, and between all factors and incidence of lung cancer: (**a**) without time lag, (**b**) lag 1 year, (**c**) lag 2 years, (**d**) lag 4 years, (**e**) lag 5 years.

Based on the BCC model, the impact of air pollution factors on the incidence of lung cancer in Nanchang was evaluated and calculated by using Matlab (R2016b version). Grey correlation analysis shows that PM_10_ is the most significant air pollution factor causing lung cancer. Consequently, the following two configurations are used to verify the effectiveness of DEA for simulating the incidence of lung cancer. The first configuration contains all the pollution factors while the second configuration contains only PM_10_. For both configurations, the time lag of the incidence of lung cancer was evaluated between none and 4 years. These results are shown in Table 7 and Figure 2.

Results show that the analyses containing all five pollution factors are very similar to the results of PM_10_. Furthermore, line graphs of the two conditions are very similar. These similarities reinforce our earlier finding that PM_10_ is the most critical air pollution factor contributing to lung cancer.

#### 3.2.2. The Relationship between the Incidence of Lung Cancer and Air Pollution for Different Types of Patients

Lung cancer patients in Nanchang during 2003 to 2014 were classified as rural or urban, male or female, and smoker or non-smoker. To further understand the relationship between the incidence of lung cancer and air pollution, these classification criteria were used simultaneously. Taking both gender and smoking history into consideration, four detailed categories were formed: male smoker (male-s), male non-smoker (male-ns), female smoker (female-s), and female non-smoker (female-ns). Using age and smoking history a further four categories were formed: smoker older than 70 (s > 70), smoker younger than 70 (s ≤ 70), non-smoker older than 70 (ns > 70), and non-smoker younger than 70 (ns ≤ 70).

The DEA method was applied to evaluate the impact of all 5 air pollution factors on the incidence of lung cancer with different time lags of lung cancer incidence ranging from none to four years. Then the patients were sorted from large to small according to their DEA effectiveness. These results are shown in Table 8.

For nearly all the time lags, DEA effectiveness suggests that urban dwellers are at the highest risk from air pollution induced lung cancer, followed by female, smoker, male, non-smoker, and rural. Table 8 shown that the DEA effectiveness between air pollution and urban patients is greater than the DEA effectiveness between air pollution and rural patients. This coincides with the incidence of lung cancer in Nanchang during 2003 to 2014.

Table 9 shown that in 2003, the incidence of lung cancer in urban areas (2.6817/100,000) was approximately 6 times the incidence of lung cancer in rural areas (0.4507/100,000). While in 2014, the incidence of lung cancer in urban areas (4.3701/100,000) and the incidence of lung cancer (4.2937/100,000) is almost identical. This change is related to the acceleration of China’s urbanisation processes and rural development.

The DEA effectiveness between air pollution and female patients is greater than the DEA effectiveness between air pollution and male patients.

The DEA effectiveness between air pollution and smoking patients is greater than the DEA effectiveness between air pollution and non-smoking patients due to smoking being one of the major causes of lung cancer. Incidence of smoking patients is approximately twice than that of non-smoking patients. Combined with the actual incidence of lung cancer, the incidence of lung cancer in both smokers and non-smokers is increasing. 

As shown in Table 8 and Table 10, for all 14 categories, the DEA effectiveness between air pollution and male smoking patients is greater than the DEA effectiveness between air pollution and male non-smoking patients. This indicates that the combined effect of smoking and air pollution exposure will greatly increase the risk of lung cancer. The incidence of lung cancer in smoking patients is more than three times that of the incidence of non-smokers with lung cancer. Considering the fact that most smokers in China are male, this phenomenon is particularly noteworthy.

The DEA effectiveness between air pollution and female non-smoking patients is greater than the DEA effectiveness between air pollution and female smoking patients. This suggests that air pollution was not significantly associated with lung cancer among female smoking patients.

## 4. Discussion

As far as we know, this study is one of the few to study the relation between air pollution and lung cancer in middle reaches of Yangtze River. By using the grey correlation analysis method and DEA, the relationship between the incidence of lung cancer and air pollution factors in Nanchang city was explored. The results obtained by the two different analysis methods were similar, which reinforces the reliability of the results.

Our study found that, by using grey correlation analysis, among five air pollution factors (annual mean concentration of SO_2_, NO_2_, PM_10_, haze days, and API/AQI), PM_10_ was the most closely related to the incidence of lung cancer in Nanchang. This result was reinforced by DEA, which showed that PM_10_ was the most critical air pollution factor for lung cancer.

One interesting finding shows that the DEA effectiveness between air pollution and urban patients was greater than the DEA effectiveness between air pollution and rural patients, which means that air pollution has more impact on urban dwellers as compared to rural dwellers. After related research of pollution sources in Nanchang, we found that automobile exhaust, soil dust, coal-burning dust, building dust and metallurgical dust are the major sources of PM_10_ in Nanchang. The main sources of pollution in urban areas are automobile exhaust emissions and industrial pollution. For rural areas, the main sources of pollution are automobile exhaust emissions, industrial pollution and pollution brought by ore (marble and limestone) mining and building stones processing [44]. Quarries, cement plants and building materials processing plants around Nanchang are some of the traditional industries that brought serious air pollution. In rural development, some out-of-date or heavy polluting industry that unable to meet environmental requirements were relocated from urban areas to rural areas in order to avoid been shut down. Some government officials welcomed these companies in order to raise GDP in their jurisdictions. Another fact that cannot be ignored was that many rural residents actually work in urban areas, they breathed the same air as the urban dwellers. All of these factors greatly increased the incidence rate of lung cancer in rural residents.

Another interesting finding is that the DEA effectiveness between air pollution and female patients was greater than the DEA effectiveness between air pollution and male patients, indicating that women are more susceptible to lung cancer caused by air pollution. For most adult Chinese women, they had to take on the responsibilities of a housewife including cooking. Unlike Western food, Chinese food uses a lot of oil in the cooking process. Hot pots and oil will produce a lot of cooking oil fumes which can potentially cause lung cancer. Several studies have shown that a significant association between cooking oil fumes and lung cancer exists for Chinese women [45,46]. Another reason is that due to the female physique is more sensitive to air pollution as compared to the male physique, women are more likely to suffer from passive smoking and cause lung cancer [45,47].

The DEA effectiveness between air pollution and smoking patients was greater than the DEA effectiveness between air pollution and non-smoking patients, meaning that smokers are more likely to cause lung cancer due to air pollution.

Industrialisation and urbanisation can promote economic development and increasing GDP growth rate. Between 2003 and 2014, Nanchang’s economy grew rapidly and GDP grew to more than five-fold within twelve years (from 64.1 billion Yuan in 2003, to 336.7 billion Yuan in 2014). However, the development of economy and the rapid growth of population have brought severe pressure to the atmosphere. As the capital city of Jiangxi Province, the problem of atmospheric pollution has become increasingly prominent in recent years. The main causes of air quality pollution are:

(1) Many enterprises have entered Nanchang, but the environmental management standards are not perfect, which makes many enterprises choose coal combustion equipment and out-of-date equipment, resulting in an increase in air pollutant emissions.
(2) The increase of population makes the real estate industry develop greatly in Nanchang, the construction of buildings blossom everywhere. Lack of dust control measures while demolishing old houses, removing wreckage and constructing new houses result in PM_10_ increased significantly. The boom in real estate industry also raises demand for more products from cement plants and quarries around Nanchang.
(3) Car ownership increased but lack of exhaust control measure. Motor vehicles with black smoke can be seen everywhere in the streets, and motor vehicle exhaust has become a major source of air pollution.
(4) The layout of industry zone is unreasonable and cannot match the meteorological condition. North industrial zone of Nanchang is located in the upper wind direction of the city's dominant wind direction, which is one of the direct reasons leading to the decline of air quality in the urban area.

According to our findings, we make the following recommendations:

(1) Priority should be given to low polluting industries to reduce the introduction of heavy polluting industries. Heavy polluting industries need to meet the environmental quality standards and pollutant discharge or emission standards before they can resume production. Increasing the usage of clean energy and renewable energy, reducing the usage of coal consumption, therefore the emission of air pollutants can greatly reduce.
(2) Strengthen the control of PM_10_ brought by real estate industry and related industries. Use water mist to reduce dust during demolition and muck trucks have to use dust cap. Encourage the use of environmentally friendly building materials. Trees and grass need to plant on open ground in order to reduce soil exposure. The pits left by quarries need more vegetation cover. By the end of 2016, the urban green coverage rate was 45%, and the greening rate of villages was 32%.
(3) The government should increase the proportion of new energy vehicles. More people should be encouraged to use public transport.
(4) Industrial areas need scientific planning to avoid areas that can affect the health of residents: an upper wind direction as the city’s dominant wind direction should be avoided, keeping a certain distance keep away from densely populated areas.

## 5. Conclusions

Air pollution is a serious problem in Nanchang. The finding shows that PM_10_ is closely related to lung cancer in Nanchang. Air pollution has more impact on urban dwellers as compared to rural dwellers. Women are more susceptible lung cancer caused by air pollution. If appropriate measures are taken, the incidence of lung cancer in Nanchang can be expected to decline in the future.

## Figures and Tables

**Table 1:** Newly diagnosed lung cancer patients in Nanchang from 2003 to 2014.

Year	Total	Residence		Gender		Smoking History	
		Urban	Rural	Male	Female	Smoking	Non-S
2003	119	99	20	84	35	77	42
2004	135	109	26	107	28	83	52
2005	102	83	19	81	21	80	22
2006	147	124	23	116	31	110	37
2007	188	160	28	140	48	132	56
2008	197	167	30	136	61	131	66
2009	202	163	39	143	59	134	68
2010	219	185	34	160	59	143	76
2011	236	179	57	170	66	146	90
2012	171	106	65	128	43	103	48
2013	446	240	206	332	114	275	171
2014	454	229	225	346	108	301	153
Mean	218	153.7	64.3	161.9	56.1	142.9	73.4
Maximum	454	240	225	346	114	301	171
Minimum	102	83	19	81	21	77	22
SD	115.6	50.6	72.1	87.0	29.4	72.2	45.3

**Table 2:** Incidence of lung cancer and air pollution situation in Nanchang from 2003 to 2014.

Year	Incidence of Lung Cancer (1/100,000)	SO_2_ (mg/m^3^)	NO_2_ (mg/m^3^)	PM_10_ (mg/m^3^)	Haze (day)	API/AQI
2003	2.682	0.051	0.034	0.100	95	82
2004	3.020	0.059	0.031	0.100	128	75
2005	2.266	0.05	0.031	0.089	133	69
2006	3.244	0.056	0.032	0.086	152	69
2007	4.104	0.054	0.034	0.083	136	67
2008	4.285	0.05	0.036	0.084	105	67
2009	4.361	0.054	0.037	0.079	84	66
2010	4.362	0.055	0.042	0.087	96	69
2011	4.654	0.056	0.038	0.089	172	70
2012	3.332	0.045	0.039	0.087	63	69
2013	8.603	0.049	0.051	0.146	109	100
2014	8.664	0.025	0.032	0.085	139	77

**Table 3:** Grey correlation for accumulated air pollution factors and accumulated incidence of lung cancer for different time lags.

Time Lag	SO_2_	NO_2_	PM_10_	Haze	API/AQI
1	0.8252	0.7955	0.8889	0.7146	0.8660
2	0.8168	0.7557	0.8966	0.6440	0.8489
3	0.8541	0.7483	0.8818	0.6013	0.8739
4	0.8423	0.7420	0.8583	0.5548	0.8338
5	0.8467	0.7934	0.8464	0.5570	0.8396
6	0.8556	0.7845	0.8351	0.5585	0.8445
7	0.8431	0.7713	0.8163	0.5526	0.8342
8	0.8060	0.7403	0.7820	0.5410	0.8030
9	0.7392	0.7395	0.7764	0.5231	0.7792
10	0.6667	0.7288	0.6804	0.5017	0.6810

**Table 4:** The incidence of lung cancer during 2010–2014 was used as a reference sequence, and the grey correlationfor air pollution factors and the incidence of lung cancer with different time lags is shown.

Time Lag	SO_2_	NO_2_	PM_10_	Haze	API/AQI
1	0.6791	0.7137	0.7716	0.6045	0.7330
2	0.7118	0.7004	0.6983	0.7311	0.7080
3	0.6836	0.7186	0.7183	0.6412	0.7052
4	0.7278	0.7377	0.7082	0.6586	0.7119
5	0.7032	0.7209	0.7087	0.6792	0.7124
6	0.6555	0.6953	0.6854	0.6631	0.6785
7	0.6951	0.6862	0.7031	0.6652	0.6917

**Table 5:** The incidence of lung cancer during 2009–2013 was used as a reference sequence, and the grey correlationfor air pollution factors and the incidence of lung cancer with different time lags is shown.

Time Lag	SO_2_	NO_2_	PM_10_	Haze	API/AQI
1	0.7908	0.8073	0.8124	0.6766	0.8249
2	0.7594	0.748	0.7627	0.6669	0.7801
3	0.7993	0.8048	0.8293	0.7048	0.8201
4	0.8145	0.8111	0.8079	0.8020	0.8232
5	0.7700	0.8109	0.7749	0.7642	0.7892
6	0.7472	0.777	0.8158	0.6408	0.7811

**Table 6:** The incidence of lung cancer during 2008–2012 was used as a reference sequence, and the grey correlationfor air pollution factors and the incidence of lung cancer with different time lags is shown.

Time Lag	SO_2_	NO_2_	PM_10_	Haze	API/AQI
1	0.8725	0.8145	0.8499	0.626	0.8695
2	0.7787	0.747	0.7919	0.6002	0.8074
3	0.6866	0.723	0.7665	0.6625	0.782
4	0.7027	0.8144	0.6499	0.7953	0.6954
5	0.8213	0.7588	0.8159	0.5155	0.7489

**Table 7:** Effectiveness of DEA for different time lags (1) between PM_10_ and the incidence of lung cancer (2) between all factors and the incidence of lung cancer.

Time Lag		2003	2004	2005	2006	2007	2008	2009	2010	2011	2012	2013	2014
None	PM_10_	0.2248	0.2531	0.2134	0.3129	0.4144	0.4276	0.4627	0.4202	0.4383	0.3567	0.4895	0.8543
	All factors	0.3984	0.3737	0.2919	0.4178	0.5444	0.6182	0.7469	0.6608	0.5909	0.7514	1	1
1 year	PM_10_		0.3082	0.2312	0.3719	0.4869	0.5268	0.5298	0.5634	0.5459	0.4246	1	0.6055
	All factors		0.4063	0.3343	0.4786	0.5866	0.5764	0.5541	0.5634	0.5458	0.4457	1	0.9332
2 years	PM_10_			0.2275	0.3257	0.4630	0.5003	0.5276	0.5214	0.5916	0.4274	0.9620	1
	All factors			0.2988	0.4670	0.5905	0.5968	0.5727	0.5430	0.5915	0.4274	1	1
3 years	PM_10_				0.3744	0.4737	0.4946	0.5033	0.5035	0.5372	0.4274	0.9841	1
	All factors				0.4572	0.5921	0.6133	0.5959	0.5776	0.6155	0.4964	1	1
4 years	PM_10_					0.3803	0.3970	0.4540	0.4700	0.5196	0.4085	1	0.9227
	All factors					0.5238	0.5998	0.6104	0.5915	0.5940	0.4690	1	0.9977

**Table 8:** Sort by DEA effectiveness of all 14 categories of patients with different time lags in descending order.

Order	No Lag	Lag 1 Year	Lag 2 Years	Lag 3 Years	Lag 4 Years
1	urban	urban	urban	urban	urban
2	female-ns	s>70	female-ns	female-ns	s > 70
3	female	male-s	female	female	female-ns
4	male-s	smoking	male-s	s > 70	male-s
5	smoking	female-ns	s ≤ 70	male-s	female
6	s ≤ 70	s ≤ 70	smoking	smoking	smoking
7	s > 70	male	male	s ≤ 70	s ≤ 70
8	ns > 70	female	s > 70	male	male
9	male	ns > 70	ns > 70	ns > 70	ns > 70
10	non-s	non-s	non-s	non-s	non-s
11	ns ≤ 70	ns ≤ 70	ns ≤ 70	ns ≤ 70	ns ≤ 70
12	male-ns	male-ns	male-ns	male-ns	rural
13	rural	rural	rural	rural	male-ns
14	female-s	female-s	female-s	female-s	female-s

**Table 9:** Incidence of lung cancer (1/100,000) in Nanchang from 2003 to 2014.

Year	Total	Residence		Gender		Smoking History	
		Urban	Rural	Male	Female	Smoking	Non-S
2003	2.6817	2.2310	0.4507	1.8930	0.7887	1.7352	0.9465
2004	3.0204	2.4387	0.5817	2.3939	0.6264	1.8570	1.1634
2005	2.2663	1.8442	0.4222	1.7997	0.4666	1.7775	0.4888
2006	3.2445	2.7368	0.5076	2.5603	0.6842	2.4278	0.8166
2007	4.1042	3.4930	0.6113	3.0563	1.0479	2.8817	1.2225
2008	4.2845	3.6321	0.6525	2.9578	1.3267	2.8491	1.4354
2009	4.3609	3.5189	0.8420	3.0872	1.2737	2.8929	1.4680
2010	4.3620	3.6848	0.6772	3.1869	1.1752	2.8483	1.5138
2011	4.6538	3.5298	1.1240	3.3523	1.3015	2.8790	1.7748
2012	3.3323	2.0656	1.2667	2.4944	0.8380	2.0072	0.9549
2013	8.6030	4.6294	3.9736	6.4040	2.1990	5.3045	3.2985
2014	8.6638	4.3701	4.2937	6.6028	2.0610	5.7441	2.9197

**Table 10:** Incidence of lung cancer among residents in Nanchang from 2003 to 2014 with different smoking history.

Year	Male		Female		Smoking		Non-Smoking	
	Smoking	Non-S	Smoking	Non-S	s >70	s ≤ 70	ns > 70	ns ≤ 70
2003	1.6676	0.2254	0.0676	0.7211	0.4732	1.2620	0.1803	0.7662
2004	1.8346	0.5593	0.0224	0.6041	0.4251	1.4319	0.2461	0.9173
2005	1.7775	0.0222	0	0.4666	0.4444	1.3331	0.1111	0.3777
2006	2.3616	0.1986	0.0662	0.6180	0.5297	1.8981	0.1986	0.6180
2007	2.8599	0.1965	0.0218	1.0261	0.7423	2.1394	0.2183	1.0042
2008	2.8274	0.1305	0.0217	1.3049	0.6960	2.1531	0.3262	1.1092
2009	2.8929	0.1943	0	1.2737	0.9283	1.9646	0.2807	1.1874
2010	2.8084	0.3784	0.0398	1.1353	0.7569	2.0914	0.4382	1.0756
2011	2.8790	0.4733	0	1.3015	0.8085	2.0705	0.4930	1.2818
2012	1.9292	0.5651	0.0779	0.7600	0.1949	1.8123	0.2728	0.6821
2013	5.1695	1.2345	0.1350	2.0640	1.0995	4.2051	0.7330	2.5655
2014	5.4960	1.1068	0.2481	1.8129	1.4503	4.2937	0.6297	2.2900
